# Supplementary material for: A conformational sensor based on genetic code expansion reveals an autocatalytic component in EGFR activation
Source: Nat Commun. 2018 Sep 21;9:3847. doi: 10.1038/s41467-018-06299-7 (PMC6155120; doi:10.1038/s41467-018-06299-7)
Supplement: Supplementary file 1 — Supplementary Information [file 41467_2018_6299_MOESM1_ESM.pdf]

Supplementary information

A conformational sensor based on genetic code expansion reveals an autocatalytic component in EGFR activation

Baumdick et al. 2018

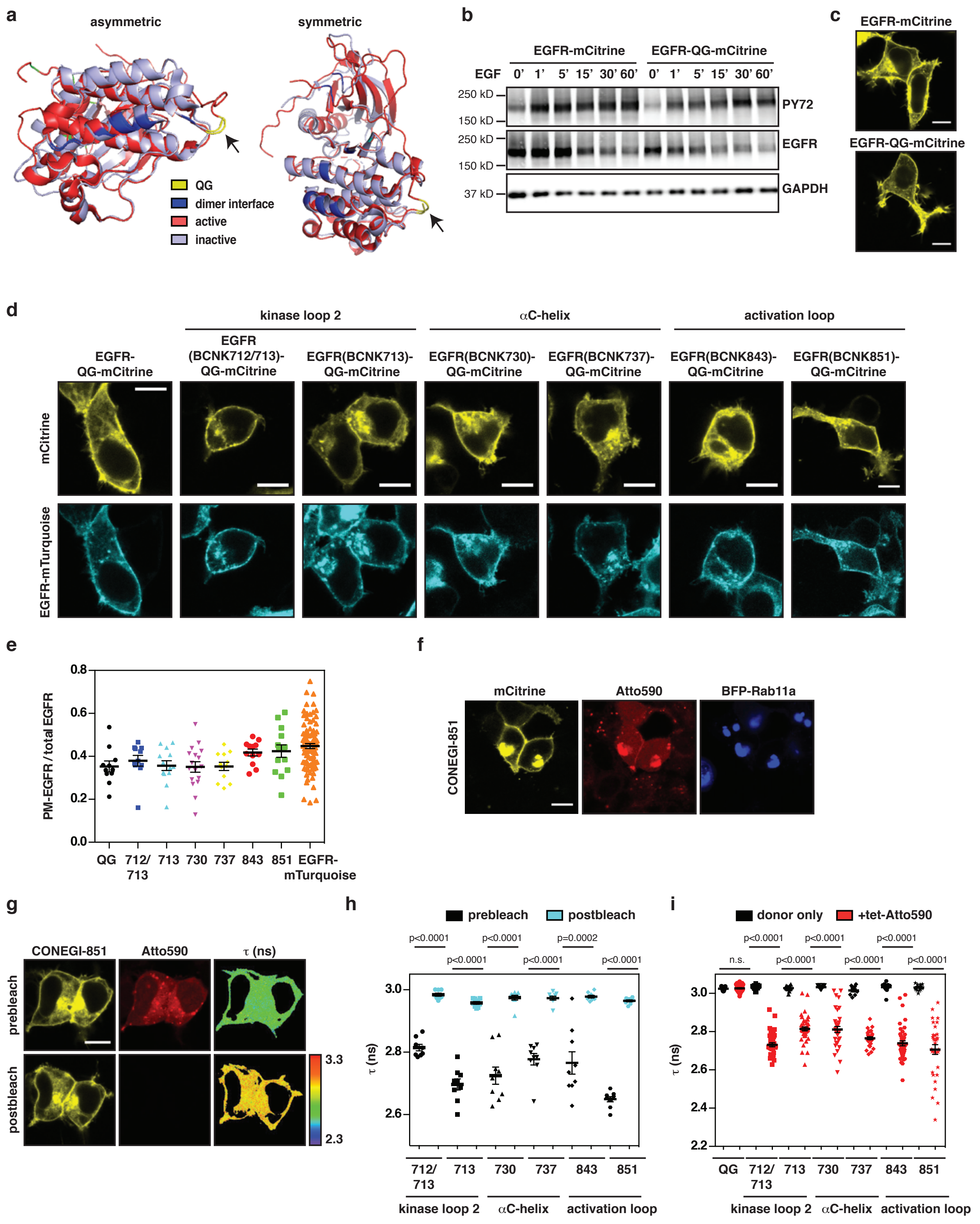

Supplementary Fig. 1. Design and characterization of CONEGI.

(**a**) Alignment of an active (red; PDB: 2J5F) and inactive (cyan; PDB: 2GS7) crystal structure of the EGFR TKD. mCitrine insertion site (yellow, black arrows) and dimerization interface (blue) of the asymmetric and symmetric dimer are indicated. (**b**) Phosphorylation of EGFR-mCitrine and EGFR-QG-mCitrine in HEK293T cells upon EGF

stimulation. Western blots using whole cell lysates were probed with anti-EGFR, anti-PY72 and anti-GAPDH (**Fig. 1c**). (**c**) Representative fluorescence images of EGFR-mCitrine and EGFR-QG-mCitrine. (**d**) Representative fluorescence images of HEK293T cells co-expressing EGFR-mTurquoise and EGFR-QG-mCitrine or EGFR(BCNKXXX)-QG-mCitrine variants. (**e**) Quantification of the relative PM-EGFR fraction (PM-EGFR/total EGFR) of EGFR-QG-mCitrine (n=11 cells), EGFR(BCNKXXX)-QG-mCitrine variants (712/713: n=11; 713: n=13; 730: n=17; 737: n=12; 843: n=12; 851: n=14) and EGFR-mTurquoise (n=90). (**f**) Representative fluorescence images of HEK293T cells co-expressing CONEGI-851 (mCitrine and Atto590 fluorescence) and BFP-Rab11a.

(**g**) Representative mCitrine and Atto590 fluorescence images of CONEGI-851 before and after photobleaching Atto590 and corresponding τ images. (**h**) Mean τ in CONEGIs (712/713: n=8 cells; 713: n=9; 730: n=9; 737: n=9; 843: n=9; 851: n=9; paired two-tailed t test) at the PM before and after photobleaching Atto590. (**i**) Mean τ of EGFR-QG-mCitrine or EGFR(BCNKXXX)-QG-mCitrine variants (QG: n=11 cells; 712/713: n=13; 713: n=12; 730: n=12; 737: n=9; 843: n=12; 851: n=14) and their corresponding CONEGI variants (QG: n=42; 712/713: n=36; 713: n=43; 730: n=36; 737: n=30; 843: n=44; 851: n=32; unpaired two-tailed t test) at the PM. Scale bars: 10 μm. EGF stimulation, 100 ng ml^{-1}. Error bars: SEM. τ, fluorescence lifetime of mCitrine.

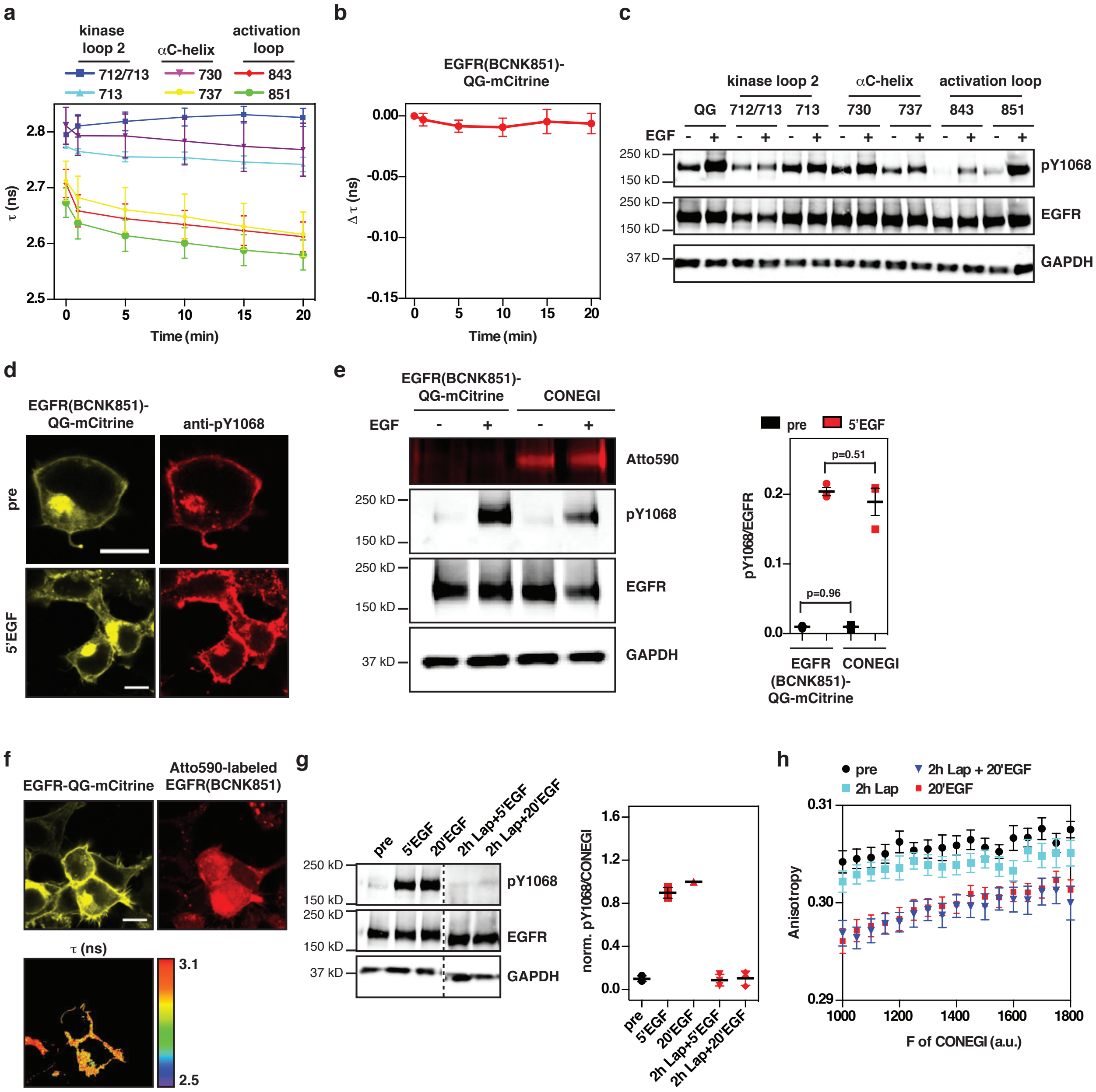

Supplementary Fig. 2. CONEGI reports on conformational transitions of the activation loop.

(**a**) Change in mean τ of CONEGI variants at the PM upon EGF stimulation (QG: n=5 cells; 712/713: n=7; 713: n=7; 730: n=7; 737: n=7; 843: n=6; 851: n=31). (**b**) Change in Δτ of EGFR(BCNK851)-QG-mCitrine at the PM upon EGF stimulation (n=3 cells). (**c**) Representative Western blot showing Y_{1068} phosphorylation of EGFR-QG-mCitrine and CONEGIs upon 5 min EGF stimulation. Blots were probed with anti-pY_{1068}, anti-EGFR and anti-GAPDH (**Fig. 2e**). (**d**) Representative fluorescence images of mCitrine and anti-pY_{1068}. HEK293T cells expressing EGFR(BCNK851)-QG-mCitrine were immunostained against pY_{1068} upon EGF stimulation (**Fig. 2g**). (**e**) Representative fluorescence image and Western blot of HEK293T cells expressing EGFR(BCNK851)-QG-mCitrine left unlabeled or 20 min labeled with tet-Atto590 show Atto590 fluorescence, Y_{1068} phosphorylation and EGFR expression upon EGF

stimulation. Blots were probed with anti-pY_{1068}, anti-EGFR and anti-GAPDH (left). Corresponding quantification of Y_{1068} phosphorylation (pY_{1068}/EGFR) of EGFR(BCNK851)-QG-mCitrine and CONEGI (n=3; unpaired two-tailed t test) (right). (**f**) Representative fluorescence images of HEK293T cells co-expressing EGFR-QG-mCitrine and Atto590-labeled EGFR(BCNK851) and corresponding τ (**Fig. 2h**). (**g**) Representative Western blot showing Y_{1068} phosphorylation of HEK293T cells expressing CONEGI upon EGF stimulation in absence or presence of 1 μM Lapatinib (Lap). Blots were probed with anti-pY_{1068}, anti-EGFR and anti-GAPDH (left). Corresponding quantification of relative Y_{1068} phosphorylation (pY_{1068}/CONEGI) of CONEGI (right) (n=3) (**h**) mCitrine fluorescence anisotropy of CONEGI versus binned mean fluorescence intensity (F of CONEGI) per pixel upon EGF stimulation in presence or absence of 1 μM Lap (N=3 experiments; n=21-26 fields of view/condition) (**Fig. 2j**). Scale bars: 10 μm. EGF stimulation, 100 ng ml^{-1}. Error bars: SEM. τ, fluorescence lifetime of mCitrine.

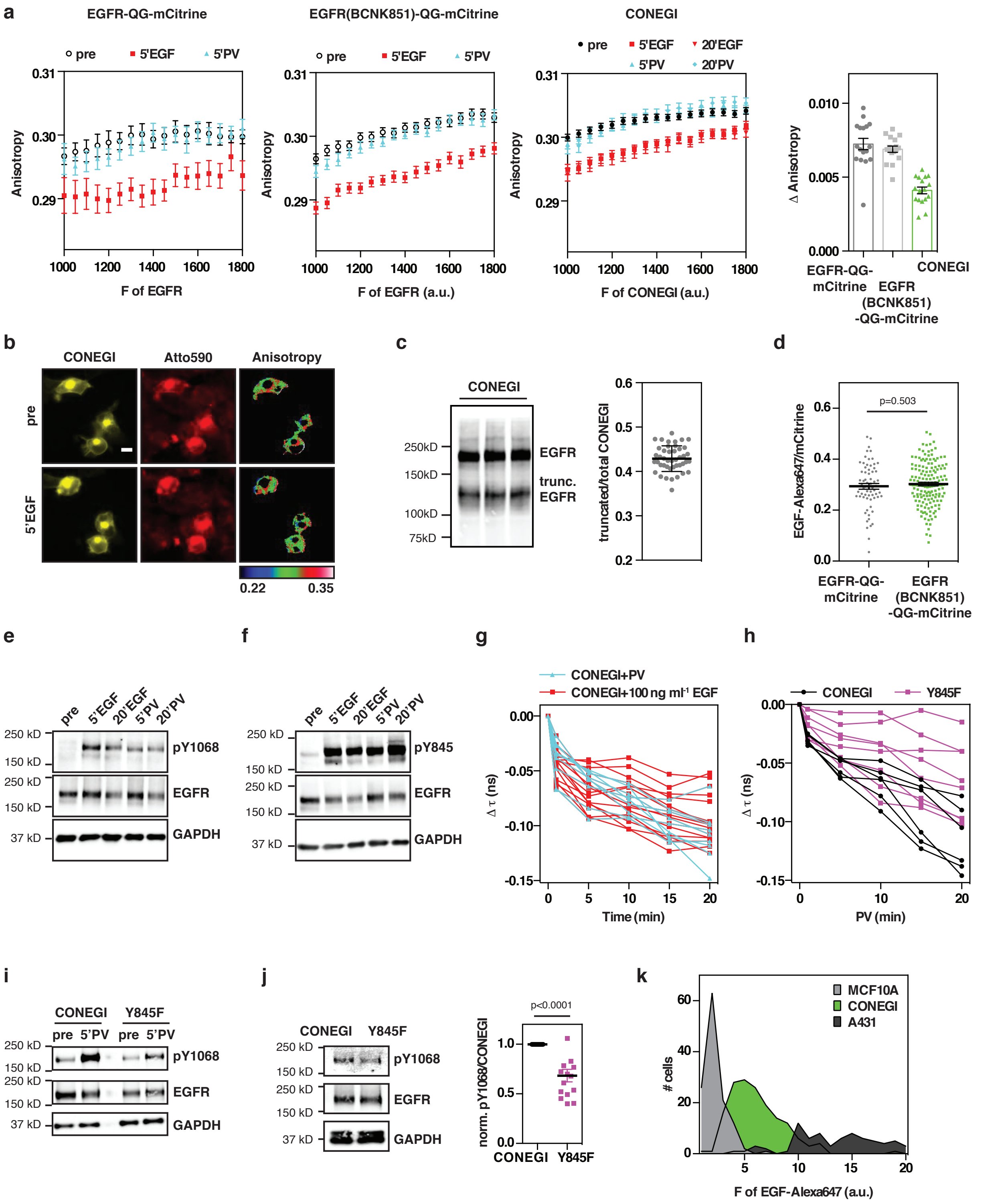

Supplementary Fig. 3. EGFR monomers phosphorylated on Y845 adopt an active conformation.

(**a**) mCitrine fluorescence anisotropy of EGFR-QG-mCitrine (left), EGFR(BCNK851)-QG-mCitrine (center left) and CONEGI (center right) versus its binned mean fluorescence intensity (F of CONEGI or EGFR-QG-mCitrine variants) per pixel in HEK293T cells upon EGF or pervanadate (PV) treatment (N=3 experiments/variant; n=25-47 fields of view/condition) (**Fig. 3a**). Change in anisotropy (Δ Anisotropy) upon 5 min EGF stimulation for EGFR-QG-mCitrine, EGFR(BCNK851)-QG-mCitrine and CONEGI (right). (**b**) Representative mCitrine and Atto590 fluorescence images

of CONEGI and corresponding mCitrine fluorescence anisotropy images upon EGF stimulation. (**c**) Representative Western blots of HEK293T lysates transfected with CONEGI showing expression of CONEGI (EGFR) and truncated CONEGI (trunc. EGFR). Blots were probed with anti-EGFR (detecting the EGFR N-terminus). (**d**) Relative ligand occupancy (EGF-Alexa647/ mCitrine) of EGFR-(BCNK851)-QG-mCitrine (n=157) and EGFR-QG-mCitrine (n=66; unpaired two-tailed t test) at the PM for individual cells upon stimulation with EGF-Alexa647. (**e,f**) Representative Western blots of HEK293T lysates transfected with CONEGI showing Y_{1068} (**e**) or Y_{845} (**f**) phosphorylation and expression of CONEGI (EGFR) upon EGF or PV treatment. Blots were probed with anti-pY_{1068} or anti-pY_{845}, anti-EGFR and anti-GAPDH (**Fig. 3b,c**). (**g**) Change in $\Delta\tau$ of CONEGI at the PM upon EGF or PV treatment in individual cells (**Fig. 3d**). (**h**) Change in $\Delta\tau$ of CONEGI and CONEGI-Y845F in individual cells upon PV treatment (**Fig. 3f**). (**i**) Representative Western blot on HEK293T lysates transfected with CONEGI or CONEGI-Y845F upon PV treatment show Y_{1068} phosphorylation and EGFR expression. Blots were probed with anti-pY_{1068}, anti-EGFR and anti-GAPDH (**Fig. 3e**). (**j**) Representative Western blot (left) on HEK293T lysates expressing CONEGI or CONEGI-Y845F show Y_{1068} phosphorylation and their expression (EGFR). Blots were probed with anti-pY_{1068}, anti-EGFR and anti-GAPDH. Corresponding normalized Y_{1068} phosphorylation (pY_{1068}/CONEGI) of CONEGI and CONEGI-Y845F in absence of ligand (right; n=15). (**k**) EGF-Alexa647 (F of EGF-Alexa647) fluorescence intensity per cell distributions of EGF-Alexa647 (F of EGF-Alexa647) bound to endogenous EGFR in MCF10A (n=125 cells) and A431 (n=129) or to CONEGI expressed in HEK293T cells (n=157). Scale bars: 10 μm. EGF stimulation, 100 ng ml^{-1}. PV treatment, 0.33 mM. Error bars: SEM. τ, fluorescence lifetime of mCitrine.

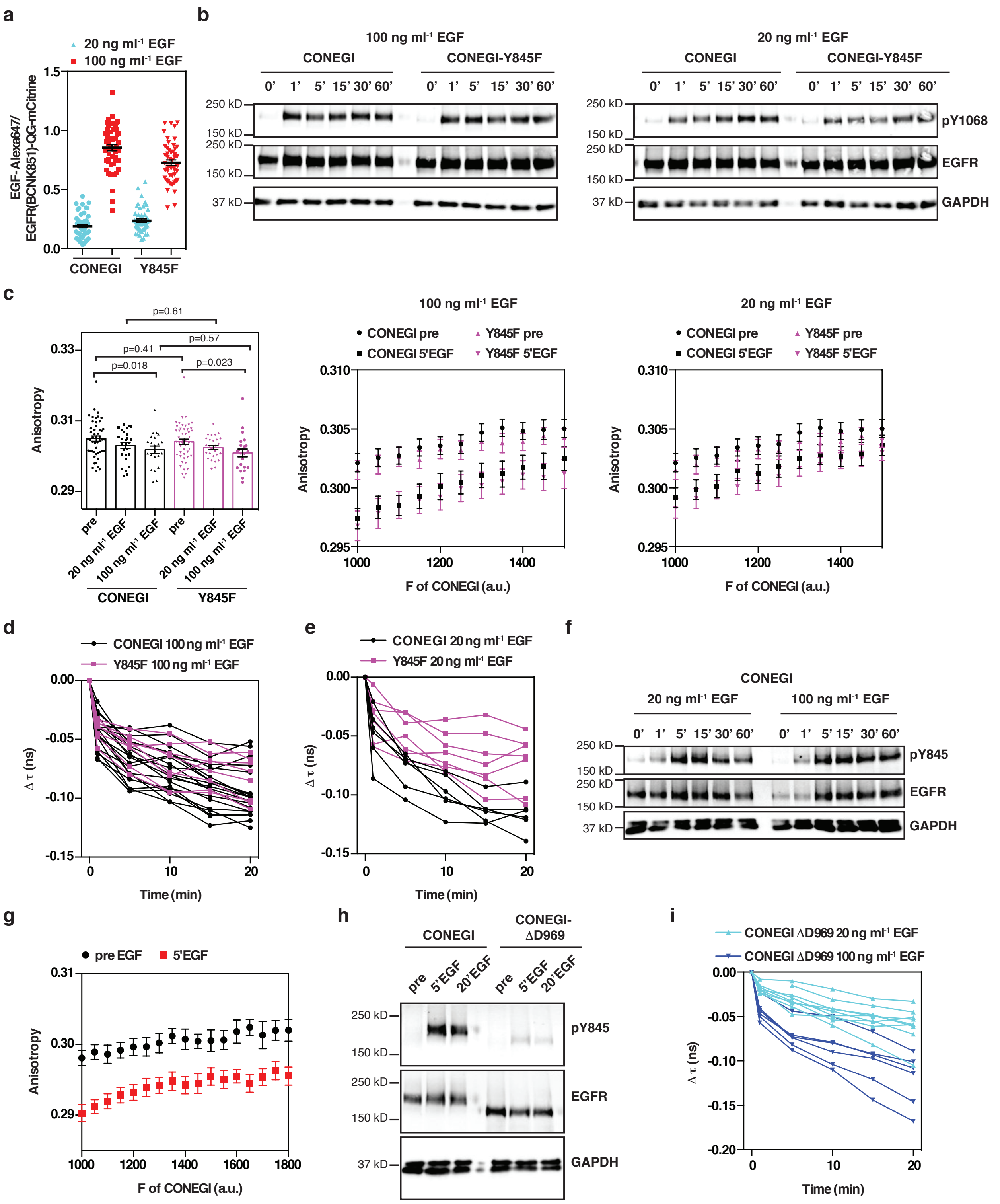

Supplementary Fig. 4. EGFR dimers can activate autocatalytic activation on EGFR monomers.

(**a**) Relative ligand occupancy (EGF-Alexa647/EGFR(BCNK851)-QG-mCitrine) of EGFR-(BCNK851)-QG-mCitrine (20 ng ml^{-1} EGF: n=52 cells; 100 ng ml^{-1} EGF: n=53) and its Y845F mutant (20 ng ml^{-1} EGF: n=50; 100 ng ml^{-1} EGF: n=50) at the PM upon stimulation with 20 or 100 ng ml^{-1} EGF-Alexa647. (**b**) Representative Western blots on HEK293T lysates expressing CONEGI or CONEGI-Y845F upon stimulation with 100 (left) or 20 ng ml^{-1} EGF

(right). Blots were probed with anti-pY_{1068}, anti-EGFR and anti-GAPDH (**Fig. 4a,c**). (**c**) Mean mCitrine fluorescence anisotropy of CONEGI and CONEGI-Y845F upon 5 min stimulation with 20 or 100 ng ml^{-1} EGF (left). mCitrine fluorescence anisotropy of CONEGI or CONEGI-Y845F versus its binned mean fluorescence intensity (F of CONEGI) per pixel in HEK293T cells before and after 5 min stimulation with 100 (middle) or 20 ng ml^{-1} EGF (right) (N=3 experiments; n=22-27 fields of view/condition; unpaired two-tailed t test). (**d,e**) Change in $\Delta\tau$ of CONEGI or CONEGI-Y845F at the PM in individual HEK293T cells upon stimulation with 100 (**d**) or 20 ng ml^{-1} EGF (**e**) (**Fig. 4b,d**). (**f**) Representative Western blot on HEK293T lysates expressing CONEGI upon stimulation with 20 or 100 ng ml^{-1} EGF. Blots were probed with anti-pY_{845}, anti-EGFR and anti-GAPDH (**Fig. 4e**). (**g**) mCitrine fluorescence anisotropy of CONEGI-ΔD969 versus its binned mean fluorescence intensity (F of CONEGI) per pixel upon stimulation with 100 ng ml^{-1} EGF (N=3 experiments). (**h**) Representative Western blot on HEK293T lysates expressing CONEGI or CONEGI-ΔD969 upon stimulation with 100 ng ml^{-1} EGF. Blots were probed with anti-pY_{845}, anti-GFP (EGFR) and anti-GAPDH (**Fig. 4f**). (**i**) Change in $\Delta\tau$ of CONEGI-ΔD969 at the PM in individual HEK293T cells upon stimulation with 20 or 100 ng ml^{-1} EGF (**Fig. 4g,h**). Error bars: SEM.

uncropped western blot and in gel fluorescence image Figure 1d:

western blot

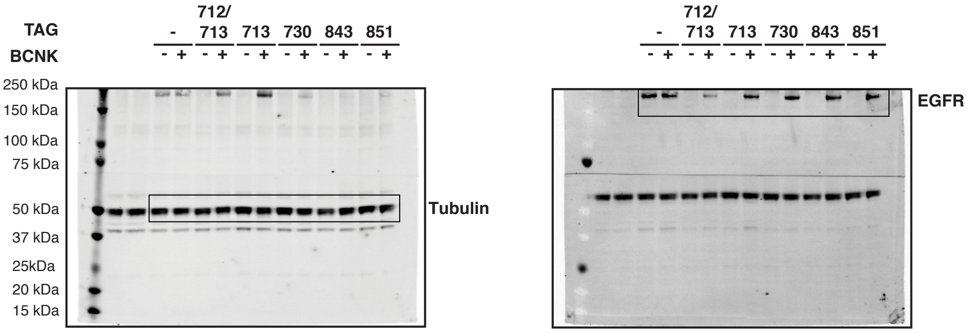

in-gel fluorescence

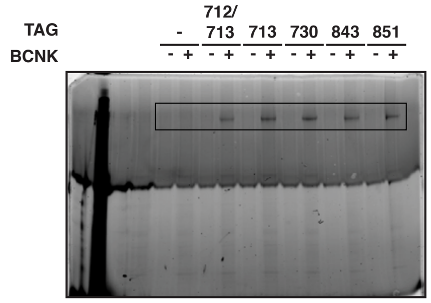

Supplementary Fig. 5. Uncropped western blots and in-gel fluorescence images.

Fluorescence images (lower row) and Western blot analysis (upper row) following SDS-PAGE of HEK293T cell lysates showing Atto590 fluorescence and expression level of EGFR-QG-mCitrine and CONEGIs depending on BCNK. Blots were probed with anti-EGFR (upper right) and anti-Tubulin (upper left).

BCNK incorporation site	Distance (Å)	Estimated FRET efficiency	Experimentally obtained FRET efficiency
712/713	80.1	0.14	0.108 ± 0.006
713	80.1	0.14	0.112 ± 0.011
730	85.2	0.1	0.105 ± 0.008
737	79.7	0.14	0.105 ± 0.007
843	81.9	0.12	0.122 ± 0.006
851	73.7	0.21	0.143 ± 0.008

Supplementary Table 1. FRET efficiencies of CONEGI variants.

Estimated distances (including linker length) between mCitrine insertion site and each BNCK incorporation site as well as estimated and experimentally obtained FRET efficiencies for each COENGI variant. To estimate FRET efficiencies the orientation factor κ^2 and the refractive index were assumed to be 2/3 and 1.4.

Plasmid		Sequence
EGFR-QG-mCitrine: HindIII insertion in EGFR	fwd	AGCGCTACCTTGTCATTCAGAAGCTTGGGGATGAAAGAATGCATTT
	rev	AAATGCATTCTTTCATCCCCAAGCTTCTGAATGACAAGGTAGCGCT
EGFR-QG-mCtirine: mCitrine linker amplification	fwd	AGCGCTACCTTGTCATTCAGCTGGCGGCGGCGTATAGCAGCATTCTGAGCAGCAACCTGAGCAGCGATAGCCGATCGATGGTGAGCAAGGGCGAGGA
	rev	AAATGCATTCTTTCATCCCCGCTGCTCAGAATGCTGCTATACGCCGCCGCCAGGCTATCGCTGCTCAGGTTCGATCGCTTGTACAGCTCGTCCATGC
EGFR(TAG712/713)-QG-mCitrine mutagenesis pcr	fwd	TCTGGATCCCAGAAGGTGAGTAGAAAGTTAAAATTCCCGTCGC
	rev	GCGACGGGAATTTTAACTTTCTACTCACCTTCTGGGATCCAGA
EGFR(TAG713)-QG-mCitrine mutagenesis pcr	fwd	TGGATCCCAGAAGGTGAGTAGGTTAAAATTCCCGTCGCT
	rev	AGCGACGGGAATTTTAACCTACTCACCTTCTGGGATCCA
EGFR(TAG730)-QG-mCitrine mutagenesis pcr	fwd	AGAGAAGCAACATCTCCGTAGGCCAACAAGGAAATCCTC
	rev	GAGGATTTCCTTGTTGGCCTACGGAGATGTTGCTTCTCT
EGFR(TAG737)-QG-mCitrine mutagenesis pcr	fwd	GCCAACAAGGAAATCCTCTAGGAAGCCTACGTGATGGCC
	rev	GGCCATCACGTAGGCTTCCTAGAGGATTTCCTTGTTGGC
EGFR(TAG843)-QG-mCitrine mutagenesis pcr	fwd	CTGCTGGGTGCGGAAGAGTAGGAATACCATGCAGAAGGA
	rev	TCCTTCTGCATGGTATTCCTACTCTTCCGCACCCAGCAG
EGFR(TAG851)-QG-mCitrine mutagenesis pcr	fwd	TACCATGCAGAAGGAGGCTAGGTGCCTATCAAGTGGATG
	rev	CATCCACTTGATAGGCACCTAGCCTCCTTCTGCATGGTA
EGFR(TAG851)-QG-mCitrine Y845F mutagenesis pcr	fwd	GGTGCGGAAGAGAAAGAATTCCATGCAGAAGGAGGCTAG
	rev	CTAGCCTCCTTCTGCATGGAATTCTTTCTCTTCCGCACC
EGFR(TAGXXX)-QG-mCitrine in (U6-PylT*)$_4$/EF1α	fwd	GGGCTAGCATGCGACCCTCCGGG
	rev	GCGCGGCCGCTCATGCTCCAATAAATTC
CONEGI ΔD969	fwd	GGGCTAGCATGCGACCCTCCGGG
	rev	GCGCGGCCGCTCATGTAGGACTTGGCAA
PTB-mCherry	fwd	CGCGAATTCATGGGCCAGCTTGGG
	rev	CGCGTCGACGTCCTGAGGTATTGTTTGAAGC
NES to (U6-PylT*)4/EF1a-PylRS: inverse pcr	fwd	ATGGACAAGAAACCCCTGGAC
	rev	TGAGCCAATTGTGAGGGTACCGAACTTCTTAGTCATCTTGTCGTCGTCGTCCTT
NES to (U6-PylT*)4/EF1a-PylRS: linker insertion	fwd	TCAGGCGGCGGCGGGTCAAGCGGCGGGGGGGGAAGCTCTGGCGGCGGTGGGTCATCCGGGGGCGGCGGCAGC
	rev	GCTGCCGCCGCCCCCGGATGACCCACCGCCGCCAGAGCTTCCCCCCCCGCCGCTTGACCCGCCGCCGCCTGA

Supplementary Table 2. List of all primers used in this study.
